# Comprehensive Modulation of Secondary Metabolites in Terpenoid-Accumulating *Mentha spicata* L. via UV Radiation

**DOI:** 10.3390/plants13131746

**Published:** 2024-06-24

**Authors:** Gaia Crestani, Kristýna Večeřová, Natalie Cunningham, Uthman O. Badmus, Otmar Urban, Marcel A. K. Jansen

**Affiliations:** 1 School of Biological, Earth and Environmental Science, Environmental Research Institute, University College Cork, North Mall Campus, T23 TK30 Cork, Ireland; 2 Global Change Research Institute, Czech Academy of Sciences, Bělidla 986/4a, 603 00 Brno, Czech Republic

**Keywords:** UV-B, secondary metabolites, photoprotection, *Mentha spicata*, essential oil, flavonoid, tocopherol, terpenoid

## Abstract

In plants, secondary metabolites change in response to environmental conditions. These changes co-regulate resilience to stressful environmental conditions, plant growth and development, and interactions between plants and the wider ecosystem, while also affecting soil carbon storage and atmospheric and climatic conditions. The objective of this study was to determine the association between UV exposure and the contents of key metabolites, including amino acids, phenolics, flavonoids, terpenoids, carotenoids, tocopherols, and phytosterols. *Mentha spicata* plantlets were grown in tissue culture boxes for 30 days and then exposed to a low dose of broadband UV-B (291–315 nm; 2.8 kJm^−2^ biologically effective UV) enriched light for eight days. Metabolite contents were quantified either immediately after the final UV exposure, or after seven days of recovery under photosynthetically active radiation. It was found that UV promoted the production of flavonoids (1.8-fold) ahead of phenolic acids (unchanged). Furthermore, the majority of monoterpenes and sesquiterpenes, constituents of valuable mint essential oil, were significantly increased through UV treatment (up to 90-fold for α-linalool). In contrast, the contents of carotenoids and tocopherols did not increase following UV exposure. A comparison between plants sampled immediately after UV exposure and after seven days of recovery showed that there was an overall increase in the content of carotenoids, mono- and sesquiterpenes, phenolics, and amino acids following recovery, while the contents of sterols and tocopherols decreased. These UV-induced changes in metabolite profile may have important consequences for agriculture, ecology, and even the global climate, and they also provide an exciting opportunity to enhance crop value, facilitating the development of improved products with higher levels of essential oils and added benefits of enhanced flavour, colour, and bioactive content.

## 1. Introduction

Secondary metabolites are specialized compounds that, amongst others, play important roles in the interactions of plants with the surrounding environment [1]. In plants, secondary metabolites comprise a wide range of structurally different molecules, including phenolics, flavonoids, terpenoids, steroids, and nitrogen or sulphur-containing compounds such as alkaloids, some of which are commercially valuable [2]. In response to environmental conditions, the contents of specific secondary metabolites can change, and understanding these environmentally induced changes is considered to be important for a variety of different reasons.

Secondary metabolites function as protectors against stress and contribute to protection against potentially harmful biotic and abiotic factors (e.g., drought, salinity, cold, UV, and herbivory) [3,4,5]. Furthermore, secondary metabolites act as antioxidants and signalling molecules [6], thus contributing to the regulation of plant growth and development in a dynamic environment [7,8]. These metabolites also contribute to interactions between plants and the wider ecosystem, for example through attracting pollinators and fruit dispersers [9], while root exudates containing secondary metabolites affect soil properties, including soil organisms and the amount of soil organic matter, a factor of considerable interest given attempts to increase soil carbon storage [10]. Volatile secondary metabolites can be released into the environment, substantially affecting atmospheric chemistry and climatic conditions [11]. Finally, plant secondary metabolites are key determinants of the commercial value of produce, determining colour, taste, odour, and nutritional and medicinal value [6,12,13,14].

Given the wide-ranging, functional importance of plant secondary metabolites, understanding the effects of environmental conditions on secondary metabolite content is crucial. Perhaps not surprisingly, the biosynthesis and accumulation of these compounds are tightly regulated via numerous environmental factors, especially light intensity and spectral composition [12].

Plants detect light through a variety of wavelength-specific photoreceptors [15], including phytochromes for red and far-red as well as blue light [16]. Additionally, cryptochromes and phototropins detect blue and UV-A radiation (315–400 nm), while UVR8 responds to UV-B wavelengths (280–315 nm) [17,18]. UVR8, in conjunction with COP1 and HY5 transcription factors, controls a variety of morpho–physiological responses to UV wavelengths up to 350 nm [17,19,20]. UVR8 activity is directly linked to the expression of key genes in the flavonoid and terpenoid pathways [21,22]. Apart from UVR8-associated changes in the secondary metabolite profile, plant responses to UV are also modulated through non-specific signalling pathways involving reactive oxygen species (ROS) and/or defence-induced hormones such as ethylene, jasmonic acid, and salicylic acid [12]. A commonly reported plant UV response is an increase in UV-absorbing, photoprotective secondary plant metabolites, such as phenolic and/or flavonoid compounds [12,16,23,24,25]. Although the UV-mediated induction of phenolic and flavonoid metabolites is well documented, much less is known about UV-mediated changes in the content of other metabolites. For example, relatively few publications have reported the effects of UV radiation on amino acids, terpenoids, carotenoids, tocopherols, and sterols [26,27,28,29,30,31,32], while some studies have reported seemingly contradictory data.

UV upregulates the expression of key genes in the phenylpropanoid biosynthetic pathway, leading to an increase in the content of phenolic acids and/or flavonoids [33,34,35]. For example, Chen et al. [36] reported upregulation of phenylalanine ammonia lyase (PAL), cinnamate 4-hydroxylase (C4H), and catechol-O-methyltransferase (COMT) in UV-exposed *Triticum aestivum* plants. Similar results were found by Inostroza-Blancheteau et al. [37] who reported enhanced gene expression of PAL, chalcone isomerase (CHS), and flavonoid 3-hydroxylase (F3′H) in UV-exposed *Vaccinium corymbosum*. However, the induction of phenylpropanoid biosynthesis genes can vary depending on UV wavelength and the specific plant organs, among other factors [38]. Therefore, the accumulation of phenolics and flavonoids is a compound-specific process that varies depending on environmental and developmental parameters. For example, Hectors et al. [30] showed a significant accumulation of kaempferol ahead of quercetin in *Arabidopsis* leaves at different developmental stages. This difference was attributed to quercetin having a higher antioxidant capacity and therefore a more important role in photoprotection against UV compared with kaempferol [39]. The biosynthesis of phenolics initiates from two aromatic amino acids, phenylalanine and tyrosine [40], which are converted to cinnamic acid and *p*-coumarate, respectively [24]. Phenylalanine ammonia lyase and the bifunctional phenylalanine/tyrosine ammonia lyase play a central role in the pathway not only because they are the first enzymes involved in the dedicated synthesis of phenolics from phenylalanine and tyrosine but also because their expression changes in response to a variety of environmental stressors [41].

The photoprotective role of phenolics is complemented with other secondary metabolites such as terpenoids [25] and particularly carotenoids [26]. Both groups of metabolites also have substantial commercial value. Flavonoids and carotenoids are extensively used as food colourants, preservatives, and antioxidants, as well as in pharmaceutical and cosmetic products [42,43] and animal feed [42], underlining their importance as commercial commodities. Carotenoids have a crucial protective function not only as pigments but also as dissipators of excess light and heat [44,45,46]. Badmus et al. [26] showed that UV induces increases in antheraxanthin, neoxanthin, violaxanthin, and lutein content in *Arabidopsis* leaves. The increase in violaxanthin has been observed in many studies, as revealed in a recent meta-analysis [47]. It is thought that other terpenoids, such as mono- and sesquiterpenes, also contribute to photoprotection, mainly as antioxidants [48], as indicated by their accumulation in response to UV [31]. Shamala et al. [22] found a time- and UV-dependent increase in volatile terpenoid content in *Camelia sinensis* plants. The study highlighted prolonged upregulation of most genes in the sesquiterpene biosynthetic pathway. In contrast, most genes in the monoterpene biosynthetic pathway seemed to be upregulated only for short periods during UV exposure [22]. However, the stimulatory effect of UV on monoterpenes and sesquiterpenes is not ubiquitous, with notable differences between different plant species [49]. All terpenoids have a common biosynthetic intermediate, mevalonic acid [50]. Interestingly, mevalonic acid is not only the precursor for the terpenoids but is also the starting point for the synthesis of sterols [51], triggering the question whether their biosynthesis is similarly enhanced through UV exposure. Sterols form a structural component of the cell membrane and also support the plant defence system in mitigating abiotic stressors, including UV [52]. This class of metabolites seems to play a protective role in conjunction with terpenoids in *Vitis vinifera* [29].

UV-mediated changes in plant metabolite profiles have not been well documented, except for the model plant *Arabidopsis thaliana*. This knowledge gap is particularly problematic in cases of plants synthesising large amounts of isoprenoid compounds, as well as commercially valuable crops with a value determined by secondary metabolites. Therefore, mint (*Mentha spicata*) was selected as the model species for this study. Mint synthesises and accumulates both mono- and sesquiterpenes, which are key components of valuable essential oil. The aims of this study were to quantify the effects of UV radiation on secondary metabolites and to determine potential interactive effects between different metabolic pathways. As UV is a particularly dynamic environmental factor, with dramatic changes in UV exposure detailed in the literature [53], we also investigated the persistence of UV effects on the plant metabolite profile. Persistence is particularly relevant in the context of harvested horticultural products that may take several days to reach either a processing facility or the consumer.

## 2. Material and Methods

### 2.1. Plant Material and UV-Exposure Stage 

*Mentha spicata* L. (Moles Seeds Ltd., Stanway, UK) seeds were sterilized and grown on agar in tissue culture boxes (RA40 plastic micro boxes, Sac 02, Deize, Belgium). The medium comprised half-strength Murashige and Skoog medium without sugar. Growth conditions comprised temperatures of 20 ± 2 °C, a relative humidity close to 100%, and 180 μmol m^−2^ s^−1^ PAR. This method of growing mint seedlings is detailed in Crestani et al. [54]. Plantlets that were 30 days old were subject to UV treatment. For this, the boxes were covered with either a UV-B blocking filter, Mylar (MY) polyester film (125 µm thickness; Tocana Ltd., Ballymount, Ireland) or a UV-B transmitting filter, cellulose acetate (CA) (95 μm thickness; Kunststoff-Folien-Vertrieb GmbH, Hamburg, Germany). Cellulose acetate also blocked UV-C radiation emitted by the UV-B lamps (TL40W/12 Philips, Eindhoven, The Netherlands). UV was applied for four hours daily between 12:00 and 16:00. The UV intensity was calculated as a daily biologically effective dose (BE) as reported in Flint and Caldwell [55]. Under CA, the BE was 2.7937 kJ m^−2^ (0.2830 kJ m^−2^ for UV-A and 2.5258 kJ m^−2^ for UV-B), while under MY, the BE was 0.3250 kJ m^−2^ (0.1797 kJ m^−2^ for UV-A and 0.1453 kJ m^−2^ for UV-B). The UV-exposure conditions used were selected as they did not result in visible plant damage, nor substantial effects on photosynthesis, whilst inducing changes in metabolite profile and plant morphology. A background of photosynthetically active radiation (PAR) was applied for 14 h from 7:00 to 21:00 at an intensity of 180 μmol m^−2^ s^−1^. The background PAR was enriched with far red to obtain a red–far red ratio of 1.6, calculated according to Franklin and Whitelam [56]. Further details about the light conditions applied during the experiment are reported in Crestani et al. [54]. All leaves were harvested immediately after the last of eight days of UV exposure, ground in liquid nitrogen, and stored at −80 °C until analysis. A total of six boxes were used for each treatment and each experimental replicate. All the compounds were analysed from five independent replicates, each made up of at least five plants per box. For simplicity, plants under MY were labelled as “−UV” while plants under CA were labelled as “+UV”.

### 2.2. Recovery Period

Following the final UV treatment, around 20 plants per treatment were transferred to John Innes II compost (William Sinclair Horticulture Ltd., Lincoln, UK) and exposed to UV-free PAR (14 h daily at an intensity of 180 μmol m^−2^ s^−1^), as reported in Crestani et al. [54], for seven days. At the end of the UV-free recovery phase, plants were rapidly frozen in liquid nitrogen and stored at −80 °C until analysis. Analysis was as described for plants harvested immediately after the final UV exposure. All the compounds were analysed from five independent replicates.

### 2.3. Amino Acid Extraction and Determination

The extraction and quantification of amino acids were performed as previously described in Crestani et al. [57]. Briefly, 100 mg of plant material previously ground in liquid nitrogen was mixed with 1 mL of 6 M HCl and incubated for 24 h at 110 °C. Evaporated samples were incubated with 1 mL of 1 M borate buffer and 2 µL diethyl ethoxymethylenemalonate (DEEM) for 50 min at 50 °C before transferring the samples to amber glass vials.

Separation of amino acids was carried out using an InfinityLab Poroshel column 120 EC—C18, 2.1 × 50 mm, 1.9 µm (Agilent Technologies, Santa Clara, CA, USA). Contents of individual amino acids were determined using an HPLC system (Agilent 1290 Infinity II LC HPLC Agilent Technologies, Santa Clara, CA, USA) connected to a diode-array detector (DAD) operating at 280 nm. To enable quantification, a standard amino acid mixture (Merck Life Science Limited, Arklow, Ireland) (Supplementary Figure S1 and Supplementary Table S1) was used at different dilutions. The content of amino acids is reported in µg g^−1^ of fresh weight (FW). For more details, see Supplementary Materials.

### 2.4. Determination of Phenolic Acids and Flavonoids 

Phenolic compounds were determined following the procedure detailed in Klem et al. [32]. About 200 mg of fresh plant material ground in liquid nitrogen was lyophilized and then extracted using 4 mL methanol–chloroform: H_2_O (1:2:2, *v*/*v*/*v*) solution. The upper polar phase was separated and used for the determination of phenolic compounds and the lower phase was used later for the determination of sterols. The separation and quantification of phenolic compounds were carried out using an UltiMate 3000 HPLC combined with an LTQ Orbitrap XL high-resolution mass spectrometer (HRMS) (Thermo Fisher Scientific, Waltham, MA, USA) with electrospray ionization and a Hypersil GOLD separation column (150 × 2.1 mm, 3 μm; Thermo Fisher Scientific). The content of phenolic acids and flavonoids is reported in peak area g^−1^ of dry weight (DW). For more details, see Supplementary Materials.

### 2.5. Determination of Phytosterols

To analyse and quantify a spectrum of phytosterols, an aliquot of the lower phenolic extraction phase was evaporated under N_2_ and derivatized at 60 °C for 30 min using 50 µL of pyridine and 70 µL of N,O-Bis(trimethylsilyl)trifluoroacetamide (BSTFA) with 1% tetramethylsilane (TMS) solution. After that, samples were again evaporated under a nitrogen stream, resuspended in 1 mL hexane, and analysed using a gas chromatograph (GC; Trace GC Ultra) coupled with a triple quadrupole mass spectrometer (MS; TSQ Quantum XLS; Thermo Scientific, Waltham, MA, USA). An Rxi-5Sil MS column (length 30 m, internal diameter 0.25 mm, film thickness 0.25 µm, Shimadzu, Kyoto, Japan) was used for separation. Cholesterol was used as an internal standard. For more details, see Supplementary Materials.

### 2.6. Determination of Monoterpenes and Sesquiterpenes

Monoterpenes and sesquiterpenes were extracted according to Večeřová et al. [58] with some modifications. Fresh plant material (500 mg) was ground in liquid nitrogen and incubated for 24 h at 4 °C in 4 mL of cold heptane. The extract was filtered to remove residual plant tissues and evaporated using a stream of nitrogen. The pellet was resuspended in 970 µL heptane, transferred to a 1.5 mL vial, and mixed with 30 µL bisabolol (Sigma Aldrich, Arklow, Ireland) with a concentration of 100 µg mL^−1^ as the internal standard. The standard was obtained via mixing linalool, α-pinene, (R)-(−)-α-phellandrene, (−)-β-pinene, myrcene, (R)-(+)-limonene, eucalyptol, ocimene, sabinene hydrate, (+)-terpinen-4-ol, (+)-pulegone, (−)-*trans*-caryophyllene, γ-terpinene, *p*-cymene, and menthol (Sigma Aldrich, Arklow, Ireland). The final concentration of the standard mix was 250 µL mL^−1^. From this stock solution, different dilutions were made to construct a calibration curve. Volatile terpenoids were separated using a Rxi-5Sil MS separation column and determined via GC-MS (Thermo Scientific, Waltham, MA, USA). Terpenoid content is reported in µg g^−1^ FW. For more details, see Supplementary Materials.

### 2.7. Carotenoid, Chlorophyll, and Tocopherol Extraction and Quantification

Carotenoids, chlorophylls, and tocopherols were quantified following [44,45,46] with some modifications. Briefly, 50 mg of mint plant material, previously ground in liquid nitrogen (and stored at −80 °C), was extracted with 400 µL 80% cold methanol (*v*/*v*) and sonicated in ice for 20 min. Then, 800 µL chloroform and 400 µL NaCl 1 M were added to the extract. After centrifugation for five minutes at 3000 rpm at 4 °C, the organic phase was transferred to a new tube and the aqueous phase was re-extracted using 400 µL chloroform. Samples were centrifuged once more at 3000 rpm for five minutes at 4 °C and the organic phases were combined and evaporated under vacuum till dryness (MiniVac evaporator, ScanVac, Labogene, Lillerød, Denmark). The pellet was resuspended in 200 µL MeOH: MTBE (60:40 *v*/*v*) and filtered with 0.22 µm syringe filters (Millipore, Bedford, MA, USA).

The content of carotenoids and chlorophylls was determined using an Agilent 1290 Infinity II LC HPLC (Agilent Technologies, Santa Clara, CA, USA) equipped with a YMC carotenoids C-30 reverse phase column 250 mm × 4.6 mm, 3 µm resin diameter (YMC Europe GmbH, Dinslaken, Germany). The standard was made up via mixing pure standards of zeaxanthin, luteolin, neoxanthin, antheraxanthin, β-carotene, α-tocopherol, ɣ-tocopherol, and δ-tocopherol (Sigma Aldrich, Arklow, Ireland). Standards were mixed, evaporated, filtered, and resuspended in MeOH: MTBE (60:40 *v*/*v*) to obtain a solution with a final concentration of 10 ppm. Carotenoids, chlorophylls, and tocopherols contents are reported in mg g^−1^ FW. For more details, see Supplementary Materials.

### 2.8. Statistical Analysis

Two-tailed *t*-tests or the nonparametric Mann–Whitney test were used to identify significant differences between the −UV and +UV plants. Data are reported as mean ± SE (Standard Error). The means were calculated from five independent replicates. All statistical analyses were performed with IBM SPSS Statistic v28 (Armonk, New York, NY, USA).

## 3. Results

Contents of amino acids, phenolic acids, flavonoids, mono- and sesquiterpenes, carotenoids, tocopherols, and sterols were quantified either immediately after the UV treatment or after seven days of recovery.

### 3.1. Amino Acids

**Figure 1:**
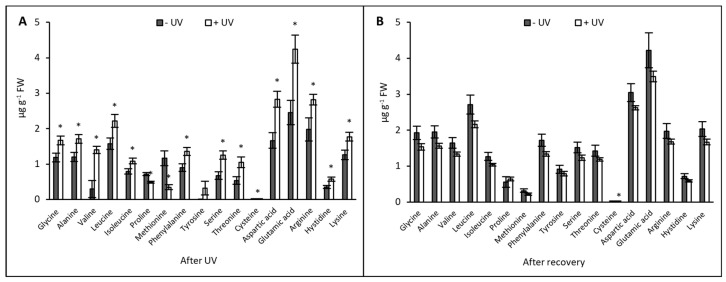
Amino acids content (µg g^−1^ FW) was measured after eight days of UV exposure (**A**) or after seven days of recovery under UV-free conditions (**B**). Grey bars represent non-UV treated plants (−UV), white bars represent UV-treated plants (+UV). Asterisks indicate a significant difference between the UV treatments with *p* < 0.05 (*). Error bars represent the SE based on the means of five independent replicates.

HPLC analyses revealed that across 17 amino acids analysed, the content of 14 amino acids had significantly increased in plants exposed to UV when measured immediately after the UV exposure (*p* < 0.05) (Figure 1A). Proline and methionine were the only two amino acids where a decrease in content was recorded in +UV plants (*p* = 0.002 & *p* = 0.016). The contents of glutamic acid, aspartic acid, and arginine were significantly increased in UV-treated plants compared with non-treated plants (*p* = 0.009, *p* = 0.006, and *p* = 0.049, respectively). Only tyrosine showed non-significant variation between the −UV and +UV plants (Figure 1A).

After seven days of recovery under UV-free conditions, the contents of most amino acids were lower in +UV plants compared with their −UV counterparts. However, these differences were not significant, except for cysteine, the content of which was significantly higher in −UV plants (*p* = 0.032) (Figure 1B).

### 3.2. Phenolic Acids and Flavonoids 

The contents of phenolic acids in leaves, such as 3-coumaric (*p* = 0.029) and syringic acid (*p* < 0.001), were significantly decreased in +UV compared with −UV plants. Also, the contents of chlorogenic acid, 3-hydroxybenzoic acid, vanillic acid, and protocatechuic acid were slightly decreased in +UV compared to −UV plants, although these differences were not statistically significant (*p* > 0.05). Ferulic acid was the only phenolic acid that increased significantly in +UV plants (*p* = 0.033). Overall, the most abundant phenolic acid was caffeic acid, which appeared not to be affected by the UV treatment and was present at almost the same level of content across both treatments. The total phenolic content also remained unchanged across −UV and +UV plants. Furthermore, the flavonoid content increased consistently 1.8-fold in +UV plants in comparison with −UV plants.

**Figure 2:**
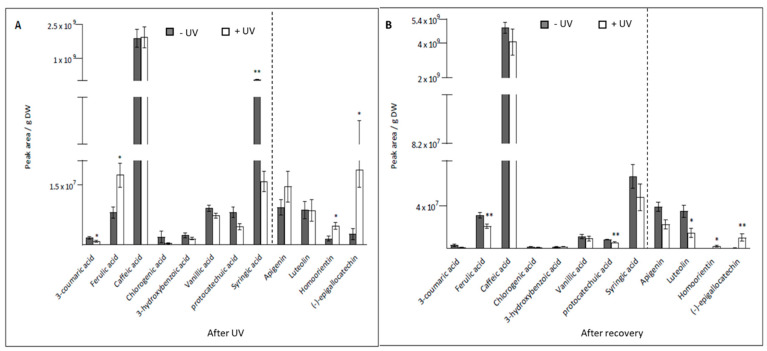
Phenolics contents (peak area/g DW) were measured after eight days of UV exposure (**A**) or after seven days of recovery under UV-free conditions (**B**). Grey bars represent non-UV treated plants, white bars UV-treated plants. The dashed lines separate phenolic acids from flavonoids. Asterisks indicate a significant difference between the UV treatments, *p* < 0.05 (*) and *p* < 0.01 (**). Error bars represent the SE based on the means of five independent replicates.

The content of specific flavonoids including apigenin and luteolin did not show any significant difference between the −UV and the +UV plants, while contents of homoorientin (*p* = 0.018) and epigallocatechin (*p* = 0.017) significantly increased following UV treatment (Figure 2A).

Variations in the contents of phenolic acid and flavonoids were also noted after seven days of recovery under UV-free conditions. The contents of ferulic acid and protocatechuic acid were significantly lower in the +UV plants that had undergone recovery compared with the −UV plants (*p* = 0.008 and *p* = 0.006, respectively). Following recovery, the contents of other phenolic acids, including 3-coumaric acid, chlorogenic acid, 3-hydroxybenzoic acid, vanillic acid, syringic acid, and caffeic acid were similar across the −UV and +UV treatments. The latter was the most abundant phenolic acid present in the mint plants. Apigenin was the only flavonoid for which no statistically significant difference was detected, but its content tended to be higher in the −UV plants after recovery. Luteolin content was significantly lower in the +UV plants (*p* = 0.02), while contents of both homoorientin and epigallocatechin were higher in the +UV plants following 7 days of recovery under UV-free conditions (*p* = 0.016 and *p* = 0.008, respectively) (Figure 2B).

### 3.3. Sterols

**Figure 3:**
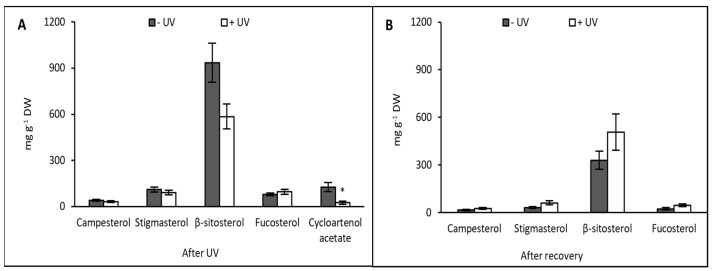
Sterol content was measured after eight days of UV exposure (**A**) or after seven days of recovery under UV-free conditions (**B**). Grey bars represent non-UV treated plants, white bars UV-treated plants. Asterisks indicate a significant difference between the UV treatments, *p* < 0.05 (*). Error bars represent the SE based on the means of five independent replicates.

The analysis of sterols revealed no overall statistical difference in contents between the non-treated and the UV-treated plants when sampled immediately after the last UV exposure. Campesterol, stigmasterol, and fucosterol were present in −UV and +UV plants in similar amounts, while β-sitosterol content was decreased but not significantly in +UV plants. Cycloartenol acetate was the only compound that was found to be significantly decreased (*p* = 0.016) in UV-exposed plants compared with non-UV-exposed ones (Figure 3A).

After seven days of recovery under UV-free conditions, the content of sterols was lower compared with samples taken immediately after UV exposure, across both −UV and +UV plants. Plants sampled after recovery showed similar content of campesterol, stigmasterol, and fucosterol, irrespective of the UV treatment. The content of β-sitosterol was higher in +UV plants after seven days of recovery on soil, but the difference between −UV and +UV was not statistically significant. Cycloartenol acetate content was below the detection limit in both −UV and +UV plants (Figure 3B).

### 3.4. Monoterpenes and Sesquiterpenes

Volatile terpenoids identified and quantified using GC-MS included 14 monoterpenes and eight sesquiterpenes. Contents of all volatile terpenoids tended to be increased in UV-exposed plants, with the exception of *p*-mentha-1,8-dien-3-one, the content of which was slightly but non-significantly lower in the UV-exposed plants. Some monoterpenes such as limonene oxide, *p*-menth-1-en-4-ol, and α-terpineol were not detected in control plants and were noted only in UV-exposed plants. Within the group of monoterpenes, significant increases in content due to UV treatment were recorded for limonene (*p* = 0.008), α-linalool (*p* = 0.008), limonene oxide (*p* = 0.008), and β-terpineol (*p* = 0.028). Although the content of sesquiterpenes was lower than that of monoterpenes, the content of the majority of sesquiterpenes was significantly increased in the plants exposed to UV. UV-treated plants accumulated significantly more sesquiterpenes such as cis-jasmone (*p* = 0.008), β-cubebene (*p* = 0.016), cadinene (*p* = 0.008), (+)-epi-bicyclosesquiphellandrene (*p* = 0.016), and germacrene D (*p* = 0.003). The contents of caryophyllene, α-farnesene, and cubenol increased but not significantly after the UV treatment. Across all the compounds analysed, the most abundant was pulegone, which was present in similar amounts in both −UV and +UV plants (Table 1).

After seven days of recovery under UV-free conditions, the content of monoterpenes was similar between the control and the +UV treatment, with the exception of myrcene, the content of which was higher but not significantly in −UV plants. Within the group of monoterpenes, the only compounds significantly different were β-pinene, α-terpineol, and *p*-mentha-1,8-dien-3-one. β-pinene was significantly higher in the plants previously exposed to UV (*p* = 0.032), while α-terpineol and *p*-mentha-1,8-dien-3-one were significantly higher in −UV plants (*p* = 0.012 & *p* = 0.005). Some monoterpenes that were not detected in the −UV treated plants immediately after UV exposure, such as limonene oxide, α-terpineol, and *p*-menth-1-en-4-ol, were found in samples that had recovered for seven days. The contents of limonene oxide and α-terpineol were slightly but not significantly higher in the −UV plants, while *p*-menth-1-en-4-ol contents were similar in the different UV treatment groups. The majority of sesquiterpenes were present at similar concentrations in −UV and +UV plants. The contents of α-farnesene, caryophyllene, and germacrene D were higher in −UV plants seven days after recovery, but not significantly so. Similar to what was noted in plants analysed immediately after the last UV exposure, pulegone remained the most abundant compound, with a similar content in −UV and +UV plants (Table 1).

### 3.5. Carotenoids, Chlorophylls, and Tocopherols

**Figure 4:**
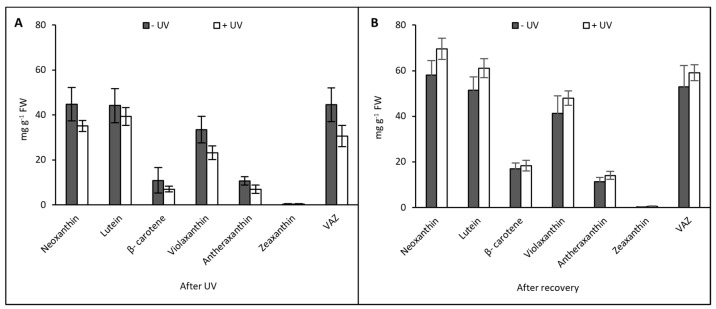
Carotenoid content (mg g^−1^ FW) was measured after eight days of UV exposure (**A**) and seven days under UV-free conditions (**B**). Grey bars represent non-UV treated plants, white bars represent UV-treated plants. No significant difference was found between the UV treatments. Error bars represent the SE based on the means of five independent replicates.

Carotenoids measured in mint leaves included neoxanthin, violaxanthin, antheraxanthin, lutein, zeaxanthin, β-carotene, and VAZ (sum of violaxanthin, antheraxanthin, and zeaxanthin). Immediately after UV exposure, no significant difference was recorded between the plants from the −UV and the +UV treatments, although a tendency for a decreased content of all carotenoids in +UV plants was noted. VAZ was decreased by ca. 33% in +UV plants, but the decrease was non-significant. (Figure 4A).

**Figure 5:**
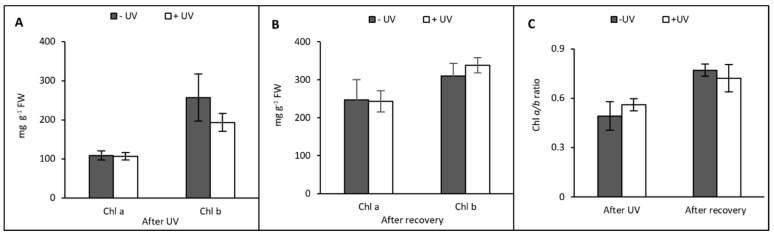
Chlorophylls *a* and *b* (mg g^−1^ FW) were measured after eight days of UV exposure (**A**) and seven days under UV-free conditions (**B**). The Chl *a*/*b* ratio was calculated after UV and after transplanting (**C**). Grey bars represent non-UV treated plants, white bars represent UV-treated plants. Error bars represent the SE based on the means of five independent replicates.

After the UV exposure, the compounds with the highest content were neoxanthin and lutein with averages of 44.80 mg g^−1^ FW and 44.20 mg g^−1^ FW, respectively. Both −UV and +UV plants had similar content of chlorophyll *a* as well as a similar chlorophyll *a* to chlorophyll *b* ratio (Chl *a*/*b*) (Figure 5). A non-significant decrease in Chlorophyll *b* content was recorded in plants exposed to UV (Figure 5A).

**Figure 6:**
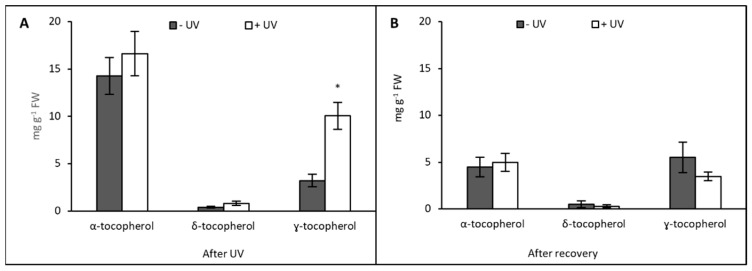
Tocopherols (mg g^−1^ FW) were measured after eight days of UV exposure (**A**) or after seven days under UV-free conditions (**B**). Grey bars represent non-UV treated plants, white bars represent UV-treated plants. Asterisks indicate a significant difference between the UV treatments, *p* < 0.05 (*). Error bars represent the SE based on the means of five independent replicates.

In contrast to carotenoids, tocopherol content tended to be increased following UV exposure. Immediately after UV exposure, the content of α-tocopherol was increased but not significantly in +UV plants compared with −UV plants. For δ-tocopherol, there was no significant difference between the −UV and the +UV plants. However, the content of ɣ-tocopherol was significantly increased following UV exposure (*p* = 0.002), reaching an average of 10.04 mg g^−1^ FW (Figure 6A). The most abundant tocopherol recorded was α-tocopherol, with an average content of 16.61 mg g^−1^ FW in UV-exposed plants.

After seven days of recovery under UV-free conditions, the overall content of carotenoids was higher compared with the previous stage. After recovery on soil, a comparison between the −UV and +UV treatments showed non-significantly higher contents of neoxanthin, violaxanthin, and lutein as well as VAZ in +UV plants. After recovery, the most abundant compound was neoxanthin, with an average of 69.57 mg g^−1^ FW in +UV plants (Figure 4B). As with carotenoids, the content of chlorophyll *a* and *b* was higher in both −UV and +UV plants compared with plants analysed immediately after the UV treatment. Similar contents of chlorophyll *a* and a similar chlorophyll *a* to chlorophyll *b* ratio (Chl *a*/Chl *b*) were found across both UV treatments (Figure 5B,C). Chlorophyll *b* content tended to be slightly higher in UV-treated plants, but the difference was not significantly different (Figure 5B).

In contrast to carotenoids and chlorophylls, the contents of all tocopherol forms were lower compared with the previous sampling stage. The content of α-tocopherol and δ-tocopherol was similar between −UV and +UV plants while ɣ-tocopherol content was higher in −UV plants, but the difference was not significant. ɣ-tocopherol was again the most abundant tocopherol, with an average content of 5.51 mg g^−1^ FW (Figure 6B).

## 4. Discussion

Previous studies have revealed that low intensities of UV radiation induce morphological and physiological changes in *Mentha spicata* [54]. These changes include a decrease in leaf area and increases in stem branching and the number of leaves. Typically, morphological changes induced via low doses of UV are not accompanied by a decrease in overall plant biomass [59]. Thus, any increase in metabolite content comprises a real increase in metabolite yield per plant, a factor that is of economical relevance, for example, where oils are extracted. Here, distinct effects of UV radiation on the accumulation of different classes of metabolites have been shown, with overall contents of amino acids, flavonoids, monoterpenes, and sesquiterpenes having increased after UV treatment. In contrast, contents of phenolics, carotenoids, chlorophylls, and sterols were mostly reduced after UV-treatment. A different trend was recorded for plants that had been recovering for seven days under PAR-only light, emphasising the transitory effect of UV on mint metabolite profiles.

### 4.1. Photoprotection: From Primary to Secondary Metabolism

UV-B induces an extensive reprogramming of plant metabolic networks [60]. For example, in the presence of UV-B, plants have been reported to upregulate the production of several amino acids [61,62,63]. This is consistent with the data obtained in this study showing increased contents of 14 amino acids after UV treatment. Amino acids are key constituents of peptides, but their role in plant cells is much wider, including functioning in a broad range of cellular processes associated with growth and development as well as stress resistance [64]. Aromatic amino acids such as phenylalanine and tyrosine can be converted into compounds such as indole alkaloids, phenylpropanoids, glucosinolates, and auxin, which are all involved in stress protection [63,65,66]. Aspartic acid and glutamic acid are not only precursors of other amino acids such as threonine, leucine, isoleucine, and methionine but are also involved in the tricarboxylic acid pathway (TCA) via their common precursor 2-oxoglutarate [67]. The metabolite 2-oxoglutarate is an important regulator and precursor for the plant’s primary and secondary metabolism, including the phenolic biosynthesis pathway [68]. Thus, the accumulation of specific amino acids is associated with a subsequent accumulation of phenolics known for their antioxidant and UV-absorbing properties, as well as overall re-programming of plant metabolism.

Our results revealed an increase in the content of most of the amino acids after UV treatment, with the exception of proline and methionine. Consistent with our results, a study conducted by Zhang et al. [69] reported that methionine was the only amino acid whose content progressively decreased under four different UV-exposure conditions. Since methionine is the key precursor of ethylene [70], this might indicate that there is a possible competition whereby the metabolite flux is directed to hormone biosynthesis and methionine is converted to ethylene. Indeed, it has been reported that ethylene content increases in UV-exposed plants [67,71]. This ethylene-mediated response is mostly associated with UV-induced stress. However, in the current study, no macroscopic signs of stress were noted, and chlorophyll and carotenoid contents remained unchanged, suggesting a mostly regulatory UV effect.

### 4.2. Redistribution of the Pool of Phenolics and Flavonoids Is Associated with Changes in Antioxidant Activity

One of the most common UV-induced responses in plants is the synthesis of phenolic acids and flavonoids that are involved in cellular protection, shielding deeper tissues of the leaf from UV penetration and participating in the antioxidant response against ROS [72,73]. Our study showed that the overall content of phenolic acid decreased after UV treatment, and this decrease paralleled an increase in flavonoids. Therefore, the question arises whether the accumulation of flavonoids rather than phenolic acids is correlated to their different role in UV protection. Csepregi and Hideg [39] showed that flavonoids have a better antioxidant capacity than phenolic acids against H_2_O_2_ and ^1^O_2_. Flavonoids’ antioxidant capacity is strictly correlated to their molecular structure and particularly the presence of a catechol group in ring B and a 3-hydroxy group and a 2,3-double bond in ring C. Consequently, plants tend to synthesise flavonoids rather than phenolic acids such as hydroxycinnamic acid, due to the superior antioxidant capacity [23]. For example, Turtola et al. [74] found that UV increased the content of flavonoids in hybrid willow plants to a greater extent than that of phenolic acids. The authors speculated that this shift was caused by UV-B enhanced flavonoid accumulation, while phenolic acids were induced in the presence of PAR-only light. In the current study, it was found that the content of phenolic acids varied in plants after UV treatment, with a tendency to be lower in plants exposed to UV, except for ferulic acid. Ferulic acid has a modest UV absorption capacity and a low antioxidant capacity [39] and also has other roles involving binding to lignin or cutin [75]. The role and rapid induction of flavonoids in response to UV have been well documented [53,76,77]. For example, Righini et al. [78] and Wang et al. [79] showed that *Arabidopsis* transgenic plants that were able to synthesise apigenin had higher protection against UV-B-induced damage. These findings, in line with our results, may be associated with the strong absorbance of apigenin in the UV range [80]. Flavonoids can be synthesized in different plant compartments, including glandular and non-glandular trichomes [81]. Glandular trichomes are also the synthesis site of other secondary metabolites such as terpenes, waxes, and lipids [82]. Even if flavonoid and terpenoid pathways are independent, they co-occur in plants to fulfil a variety of different functions in plant–environment interaction [83]. An intriguing question is whether terpenoid accumulation affects flavonoid accumulation in plants such as mint.

### 4.3. UV Enhances the Synthesis of Short-Chain Rather Than Long-Chain Terpenoids

The genus *Mentha* includes a wide number of species and hybrids with hundreds of subspecies and cultivars [84]. Due to the presence of different species and varieties, the composition of the essential oil (EO) is extremely variable [85]. Furthermore, the chemical diversity of EO is also subject to variation under different environmental conditions [86]. Essential oils consist of a mixture of different compounds with a volatile nature and include large amounts of monoterpenes and sesquiterpenes [85]. Although the quality of EO cannot be assessed through terpene contents alone, our results showed that the application of UV-B resulted in changes in the composition of the terpene pool in the essential oil, inducing an overall accumulation of mono- and sesquiterpenes immediately after UV exposure. A likely explanation for the accumulation of these compounds could be the direct effect of UV on the development of glandular trichomes, specialized structures on the leaf where the EO is stored and synthesized [87]. Ioannidis et al. [88] reported that the application of UV-B increased the oil content in the glandular trichomes in *Ocimum basilicum*. Other studies also reported an effect of UV on glandular trichomes’ size and density in *Artemisia annua* and *Betula pendula*, respectively [89,90,91]. In contrast, Escobar-Bravo et al. (2019) [91] reported an absence of correlation between UV exposure and an increased number of glandular trichomes and variation in the EO content in tomato plants, suggesting a possible species-dependent response. Our data demonstrate that there was not only a quantitative variation in the content of mono- and sesquiterpenes but also that the composition of mono- and sesquiterpenes varied under UV. The mechanism that leads to the synthesis of volatile terpenes needs to be clarified, but the stimulatory effect of UV on mono- and sesquiterpenes has been widely reported across different aromatic plant species [31]. In particular, a recent study conducted on basil found an increase in numerous monoterpenes including pinene, terpineol, and linalool [14]. In contrast, Llusia et al. [92] found more variable results whereby UV either induced or lowered terpene content depending on the plant species. Our data showed that sesquiterpene content in spearmint increased immediately after UV-B exposure. These data match the findings of Maffei and Scannerini [93] and Dolzhenko et al. [28], who noted an increase of germacrene D and β-caryophyllene in peppermint plants after UV exposure. The authors reported that the increase of sesquiterpenes was likely to have been associated with an increase of farnesyl pyrophosphate (FPPS) gene expression that was highly upregulated in the indoor UV-treated plants compared with the outdoor UV-treated plants [28].

Other secondary metabolites, including carotenoids, tocopherols, chlorophylls, and hormones such as gibberellins, are synthesised via the same precursors as monoterpenes [94]. Carotenoids are known to be strong antioxidants and are involved in plant photoprotection, playing a central role in the dissipation of excessive light energy [95]. Many studies have found a stimulatory effect of UV on carotenoids, suggesting a conserved response [6,26,44,45,96]. However, the results of this study showed that carotenogenesis was not affected immediately after the application of a low dose of UV. Thus, it is argued that carotenoids are not involved in the short-term acclimatory response against low doses of UV in mint. Nazari and Zarinkamar [97] reported a reduction in carotenoids in *Mentha aquatica* exposed to UV-B. Those authors speculated that UV might degrade carotenoids and chlorophylls or, alternatively, affect their biosynthesis or the biosynthesis of their precursors. As the content of their precursor (i.e., mevalonic acid) also increases, it is more likely that UV controls the terpene pathway with a tendency to synthesise mono- and sesquiterpenes rather than carotenoids and that this mechanism is species-specific. Due to the overlap of the biosynthetic pathways, it is speculated that mint plants exposed to UV prioritize the synthesis of shorter terpenoids, redirecting resources to other metabolic pathways (Supplementary Table S2).

### 4.4. UV Modulates Accumulation of Tocopherols and Phytosterols

In conjunction with carotenoids, tocopherols are reported to fulfil the role of antioxidants and take part in the protection of plants against environmental stressors [98]. Tocopherols partially share their biosynthetic pathway with terpenoids, which provides the isoprenoid chain, and also with the shikimate pathway from which the aromatic ring is derived [98]. Our results showed an overall increase in tocopherol content immediately after UV exposure, with the increase in γ-tocopherol being statistically significant (Figure 6). However, Munné-Bosch and Alegre [99] reported an increase in α-tocopherol. Such an increase may be associated with the plant’s response to UV-related damage to the chloroplast membranes and is often associated with stress tolerance. In line with our results, Emiliani et al. [46] also found a significant increase in ɣ-tocopherols, and this was associated with an increase in carotenoids in *Arabidopsis* plants. The authors conclude that the increase in tocopherol can contribute to UV-B protection but does not play a central role in plant protection. Moreover, DeLong and Steffen [100] conducted a study on spinach thylakoids and showed that α-tocopherol was involved in the antioxidant response against lipid peroxidation. In the same way, Badmus et al. [27] reported a time-dependent accumulation of tocopherols in *Arabidopsis* plants exposed to UV. However, other studies reported that tocopherol levels tend to be lowered following UV treatment [100,101,102]. The variation in tocopherol content might depend primarily on the presence of other antioxidants produced by the plant to counter ROS and avoid lipid peroxidation in the chloroplasts [99]. Thus, it has been hypothesised that in mono- and sesquiterpene-accumulating mint plants, the role of tocopherols is not as pronounced as reported for *Arabidopsis* by Badmus et al. [27].

Phytosterols are also involved in stress protection. Indeed, studies have reported an overall accumulation of sterols under UV [51]. For example, a paper by Shahzad et al. [103] reported that an exogenous application of β-sitosterol offered protection against UV. Our results show that the levels of some sterols remained unaffected by UV, but others showed a marked decline immediately after UV treatment, such as β-sitosterol and cycloartanol acetate. Gil et al. [29] found that changes in the content of β-sitosterol in grapevine leaves were UV-dose-dependent. In fact, low doses of UV enhance the synthesis of β-sitosterol while high doses of UV induce the synthesis of other protective metabolites that originate from the same metabolic pathway, such as mono- and diterpenes [29]. Thus, it appears that results need to be interpreted in the context of the UV dose used. Cycloartenol is one of the precursors of other sterols, including β-sitosterol, stigmasterol, and campesterol [104]. It has been reported, by Du et al. [52], that reduced activity of cycloartenol synthase, a key enzyme in the synthesis of sterols, can cause phenotypic alterations in plants. Indeed, mint plants exposed to UV showed an altered phenotype with more compact plants and more branches [54]. It is most likely that fluctuations in sterol composition are associated with their primary role in regulating plant growth and development [51] rather than being involved in plants’ stress protection.

### 4.5. Persistent and Transitory Effects of UV on Metabolite Contents

The application of UV as a tool to modulate the content of individual classes of metabolites, and especially flavonoids, is well established. However, the effects of UV on other classes of metabolites, including amino acids, terpenoids, tocopherols, and phytosterols, are still largely unknown. Also largely unknown is the persistence of UV-induced change in the metabolite profile. Nevertheless, this is an important aspect given the highly dynamic character of solar UV exposure [53]. The persistence of UV-induced changes in the metabolite profile is also critical for horticultural applications. Depending on the specific crop, produce may wait several days between harvest (and last UV exposure) and the consumer [105]. Furthermore, produce can be exposed during that period to strikingly different environmental conditions, for example, drought in an air-conditioned truck. Thus, for any UV effect to be of benefit to consumers, induced metabolites need to be stable for several days. A limited number of studies have explored the persistence of UV-induced changes after cessation of UV treatment, in what is often referred to as a “recovery period”. For instance, Hectors et al. [30] showed fluctuations in different classes of secondary metabolites in *Arabidopsis thaliana* plants after a 20 h recovery period. Specifically, the content of both flavonoids and α-tocopherol increased while that of polyamines decreased after the recovery period. In contrast, Lidon and Ramalho [106] explored the dynamic effects of a two-week recovery period on *Oryza sativa*. On the final day of recovery, it was found that VAZ content was significantly increased in UV-B exposed plants compared with control plants, while α and β-carotene contents were similar to those in the control [106]. In the current study, a comparison between the plants sampled immediately after UV exposure and after seven days of recovery showed that there was an overall retention, or even enhanced increase, of the content of economically valuable carotenoids, mono- and sesquiterpenes, phenolics, and amino acids at the end of the recovery phase, while contents of sterols and tocopherols decreased. Thus, the effects of UV exposure persist for at least one week, with potential benefits for human consumers. However, as indicated in the metabolite retention data, precision manipulation is required. In order to obtain biomass enriched in sterols and tocopherols, recent UV exposure is necessary. Conversely, where biomass enriched in carotenoids, mono- and sesquiterpenes, phenolics and amino acids is sought, UV treatments as long ago as seven days are acceptable. Metabolite-focussed precision manipulation creates novel opportunities for pre-harvest, and potentially post-harvest, UV applications that improve the secondary metabolite profile in a cost-effective and targeted manner. The expansion of indoor and urban farming, together with the rapid development of UV-emitting LEDs, opens a new window of opportunity in crop manipulation.

## 5. Conclusions

This study demonstrated comprehensive changes to the plant metabolite profile in response to UV exposure. It was found that UV promoted the production of flavonoids ahead of phenolic acids, while most amino acids, monoterpenes, and sesquiterpenes were also highly induced via UV treatment. Analysis of plants after seven days of recovery showed that increased contents of carotenoids, mono- and sesquiterpenes, phenolics, and amino acids persisted after cessation of UV treatment. In turn, these changes in metabolite profile can affect resilience to stressful environmental conditions, plant growth and development, interactions between plants and the wider ecosystem, soil carbon storage, and atmospheric and climatic conditions. In this context, this study has demonstrated that UV can be used as a powerful tool to boost the commercial value of a crop species. In mint, UV induces key components of flavour and colour, including terpenoids, that are widely used in the pharmaceutical, cosmetic, and food industries. Furthermore, the effects of UV on terpenoids persist after the end of the UV treatment; therefore, UV application can directly benefit both processors and consumers.

## Figures and Tables

**Table 1:** Contents of volatile terpenoids (µg/g FW) were measured after eight days of UV treatment or after seven days of recovery under UV-free conditions in non-UV-exposed plants (−UV) and in UV-exposed plants (+UV). Mono- and sesquiterpenes are ranked according to increasing retention time. B.D. indicates that the content was below the detection limit. Asterisks indicate a significant difference between the UV treatments, *p* < 0.05 (*) or *p* ≤ 0.01 (**). Non-significant differences between treatments are identified as n.s. The mean from five independent replicates is reported ± S.E.

		After UV Treatment			After Recovery		
Terpenes	Class	−UV	+UV	Sig.	−UV	+UV	Sig.
*α-pinene*	Monoterpene	0.029 ± 0.019	0.068 ± 0.006	n.s.	0.124 ± 0.029	0.166 ± 0.1222	n.s.
*Thujene*	Monoterpene	0.025 ± 0.024	0.078 ± 0.010	n.s.	0.147 ± 0.030	0.220 ± 0.160	n.s.
*β-pinene*	Monoterpene	0.059 ± 0.052	0.182 ± 0.253	n.s.	0.402 ± 0.076	0.520 ± 0.392	*
*Myrcene*	Monoterpene	1.007 ± 0.926	3.557 ± 0.601	n.s.	7.440 ± 1.345	10.082 ± 7.457	n.s.
*Limonene*	Monoterpene	0.135 ± 0.042	1.753 ± 0.478	*	3.764 ± 0.638	4.063 ± 2.915	n.s.
*Eucalyptol*	Monoterpene	0.176 ± 0.077	0.198 ± 0.120	n.s.	0.79 ± 0.360	0.745 ± 0.460	n.s.
*α-linalool*	Monoterpene	0.009 ± 0.006	0.815 ± 0.437	*	0.327 ± 0.041	0.313 ± 0.140	n.s.
*Limonene oxide*	Monoterpene	B.D.	0.178 ± 0.033	*	0.392 ± 0.085	0.257 ± 0.155	n.s.
*β-terpineol*	Monoterpene	0.015 ± 0.007	0.074 ± 0.020	*	0.172 ± 0.040	0.135 ± 0.039	n.s.
*p-menth-8-en-3-one*	Monoterpene	2.157 ± 0.760	2.858 ± 0.712	n.s.	3.050 ± 0.623	1.991 ± 0.815	n.s.
*p-menth-1-en-4-ol*	Monoterpene	B.D.	0.032 ± 0.015	n.s.	0.055 ± 0.012	0.065 ± 0.021	n.s.
*α-terpineol*	Monoterpene	B.D.	0.855 ± 0.429	n.s.	6.220 ± 1.611	3.956 ± 1.011	*
*Pulegone*	Monoterpene	20.33 ± 6.973	21.68 ± 4.586	n.s.	23.389 ± 4.761	22.642 ± 4.564	n.s.
*p-mentha-1,8-dien-3-one*	Monoterpene	5.675 ± 4.510	3.828 ± 1.591	n.s.	15.882 ± 2.687	11.832 ± 2.368	**
*Cis-jasmone*	Sesquiterpene	0.327 ± 0.222	2.115 ± 0.379	*	3.916 ± 0.949	3.401 ± 0.458	n.s.
*β-cubebene*	Sesquiterpene	0.010 ± 0.010	0.087 ± 0.013	*	0.096 ± 0.027	0.113 ± 0.014	n.s.
*Caryophyllene*	Sesquiterpene	0.060 ± 0.044	0.256 ± 0.068	n.s.	0.430 ± 0.146	0.133 ± 0.03	n.s.
*Cadinene*	Sesquiterpene	0.013 ± 0.008	0.088 ± 0.011	*	0.085 ± 0.013	0.058 ± 0.007	n.s.
*α-farnesene*	Sesquiterpene	0.030 ± 0.030	0.115 ± 0.017	n.s.	0.306 ± 0.087	0.149 ± 0.013	n.s.
*(+)-epi-bicyclosesquiphellandrene*	Sesquiterpene	0.018 ± 0.018	0.117 ± 0.012	*	0.169 ± 0.036	0.131 ± 0.023	n.s.
*Germacrene D*	Sesquiterpene	0.198 ± 0.061	0.718 ± 0.106	*	1.205 ± 0.204	0.911 ± 0.098	n.s.
*Cubenol*	Sesquiterpene	0.060 ± 0.018	0.120 ± 0.013	n.s.	0.164 ± 0.042	0.182 ± 0.021	n.s.

## Data Availability

Data are contained within the article and Supplementary Materials.

## Supplementary Materials

The following supporting information can be downloaded at: https://www.mdpi.com/article/10.3390/plants13131746/s1.
