# Supplementary figures and images for: Comprehensive Modulation of Secondary Metabolites in Terpenoid-Accumulating Mentha spicata L. via UV Radiation
Source: Plants (Basel). 2024 Jun 24;13(13):1746. doi: 10.3390/plants13131746 (PMC11243551; doi:10.3390/plants13131746)

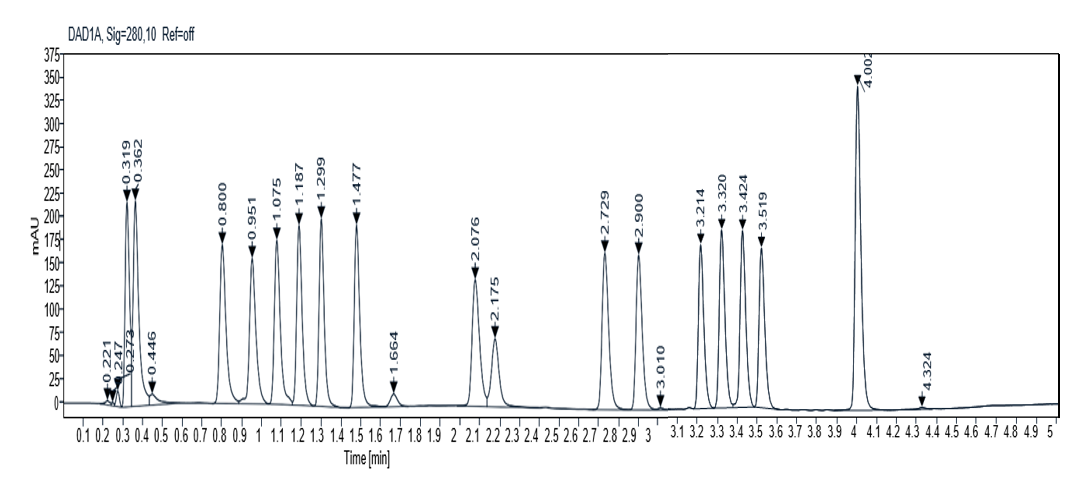

Supplement: Supplementary file 1 [file plants-13-01746-s001.zip › Supplementary Figure S1.png]

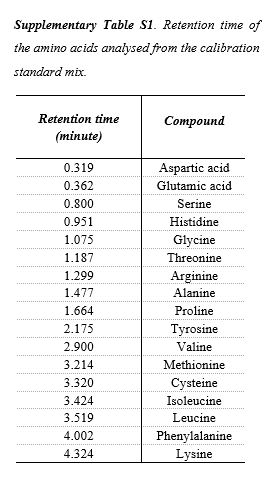

Supplement: Supplementary file 1 [file plants-13-01746-s001.zip › Supplementary Table S1.JPG]

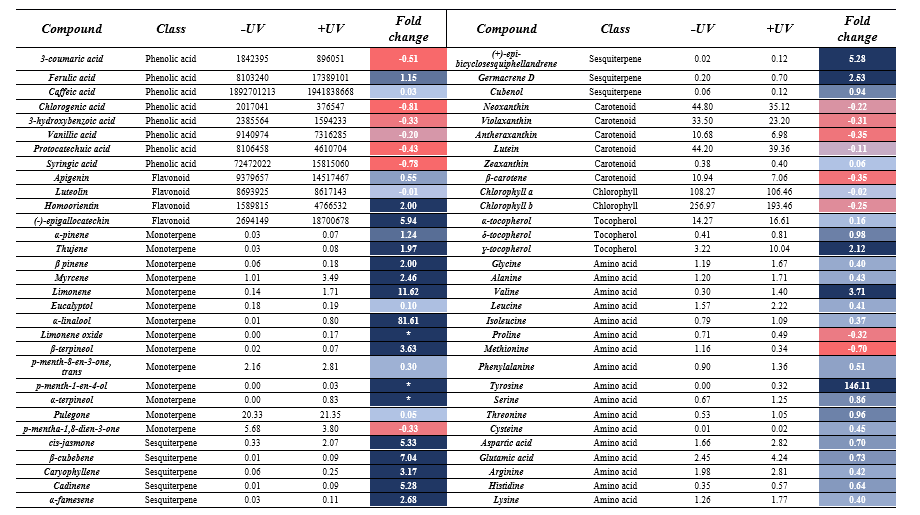

Supplement: Supplementary file 1 [file plants-13-01746-s001.zip › Supplementary Table S2.PNG]
